# Pregnancy Complicated by Ovarian Tumours

**Published:** 1908-04-25

**Authors:** 


### Obstetrics.

### PREGNANCY COMPLICATED BY OVARIAN TUMOURS.

The coexistence of ovarian tumours and pregnancy is comparatively common, and often gives rise to difficulties of diagnosis, if not of treatment. In some instances diagnosis is not difficult; at least, it is not difficult to decide that pregnancy and a tumour coexist, though it may not be easy to say exactly what the tumour is. If it appears to be of such a nature and in such a position that it will interfere with the natural course of labour, this diagnostic difficulty does not influence the treatment; for it is clear that an exploratory operation is imperative, and whatever is found must be surgically dealt with, so that labour may at least have a chance to terminate in the patient's favour.

The discovery of a tumour complicating pregnancy is nearly always accidental, the patient seeking advice for some pain or disturbance which is not usually associated with pregnancy. The diagnosis of pregnancy in such a case will depend upon the common signs. Amenorrhœa is very uncommon in uncomplicated ovarian tumours, except in bilateral disease, in which contingency both ovaries may be destroyed. If pregnancy is very early, the usual difficulty of making a sure diagnosis will be present, but there can be no doubt that the smaller the uterus is, the more readily can any tumour be felt. When the pregnancy is far advanced, if the ovarian tumour is small, it becomes correspondingly more difficult to distinguish. In a classical case two tumours should be obviously present, and a physical examination should divulge which is the pregnant uterus. If the fœtal heart can be heard over one tumour and not over the other, the diagnosis becomes the more easy. A considerable proportion of the recorded cases of ovarian tumours with pregnancy have been dermoids, and hence are small tumours and sometimes adherent from old peritonitis. Adhesion of a small tumour deep in the pelvis proves an insuperable bar to normal delivery, and gives rise to great difficulties in diagnosis. Another difficulty that may arise is torsion of the pedicle of an ovarian cyst with a pregnant uterus—an accident very likely to occur as a direct result of the uterine enlargement. In a case within the writer's experience this accident happened to a large ovarian cyst, the patient being in the fourth month of her pregnancy. The usual signs of pregnancy were present, but the physical examination of the abdomen showed enormous enlargement, the girth being considerably greater than that usual at the full term of pregnancy. This enlargement was clearly due to an accumulation of fluid, but at the time when the examination was made it was impossible to say whether this fluid was free or whether it was in the uterus—*i.e.* that the case was one of acute hydramnios. The latter diagnosis was somewhat supported by the presence of a small amount of albumen in the urine. However, on palpating through this fluid mass, something hard could just be touched a little above the umbilicus. This was clearly too high up for a pregnant uterus of four months, and from the clear history of four months' amenorrhœa it seemed unlikely that the pregnancy was further advanced than this. This hard structure could only just be reached, and its contour could not in any way be made out. Upon opening the abdomen, fifteen pints of free ascitic fluid were withdrawn, and a large ovarian tumour attached to a pregnant uterus, whose size corresponded to four months' amenorrhœa, was found. This tumour was dark purple in colour and its pedicle was tightly twisted; it was easily removed after ligaturing the pedicle, and the patient recovered without any interruption of the pregnancy. The cyst was of the ovarian multilocular type, and its pedicle had become twisted as a result of pregnancy, followed by an infection of the peritoneum, leading to the enormous outpouring of free ascitic fluid. Such a case presents insuperable difficulties to exact diagnosis, but, fortunately, as the correct line of treatment was never in doubt, a happy ending to the case was achieved.

In practice then, we must admit that there is no symptom which will make us suspect the coexistence of a tumour with pregnancy, and the patient will be fortunate who is submitted to an examination on some other grounds. The procedure in such a case will naturally include a careful survey of the history first, and in this the signs of pregnancy will be elicited. Then a complete examination of the breasts, abdomen, and pelvis must be made to discover: (*a*) if pregnancy exists, (*b*) if it is normal or complicated. The presence of an ill-defined mass in the pelvis, difficult to distinguish from the enlarging uterus, is a condition which may require several examinations at intervals of, perhaps, a fortnight or a month before its real nature can be determined. A very important help in such a case, if pregnancy is far advanced, is the fact that a pelvic tumour prevents the fœtal head and uterus descending normally into the pelvic brim. The consistence of the tumour, and perhaps fluctuation in it, may help to distinguish it from the uterus or fœtal parts. When the diagnosis is made, the treatment to be adopted is rarely in doubt. If the tumour is pelvic and will interfere with labour, it must be removed at once. It is usually taught, too, that any ovarian cyst discovered during pregnancy should be removed at once, on account of the possibility of torsion of the pedicle or rupture of the cyst. If the tumour will not interfere with labour and is not so large as to distend injuriously the abdomen, it might be left until labour is ended, but this is not recommended. The removal of an ovarian cyst during pregnancy involves no extra risk, and in the majority of cases does not result in abortion. It is advisable, however, in performing the operation, to interfere as little as possible with the pregnant uterus. If abortion should occur, it presents no more risk after an ovariotomy than at any other time.

